# Bacterial Cellulose—A Remarkable Polymer as a Source for Biomaterials Tailoring

**DOI:** 10.3390/ma15031054

**Published:** 2022-01-29

**Authors:** Lăcrămioara Popa, Mihaela Violeta Ghica, Elena-Emilia Tudoroiu, Diana-Georgiana Ionescu, Cristina-Elena Dinu-Pîrvu

**Affiliations:** Department of Physical and Colloidal Chemistry, Faculty of Pharmacy, Carol Davila University of Medicine and Pharmacy Bucharest, 6 Traian Vuia Str., 020956 Bucharest, Romania; lacramioara.popa@umfcd.ro (L.P.); elena-emilia.tudoroiu@drd.umfcd.ro (E.-E.T.); diana.ionescu@drd.umfcd.ro (D.-G.I.); cristina.dinu@umfcd.ro (C.-E.D.-P.)

**Keywords:** bacterial cellulose, ecological biopolymer, composites, biomedical applications, tissue engineering, wound dressing

## Abstract

Nowadays, the development of new eco-friendly and biocompatible materials using ‘green’ technologies represents a significant challenge for the biomedical and pharmaceutical fields to reduce the destructive actions of scientific research on the human body and the environment. Thus, bacterial cellulose (BC) has a central place among these novel tailored biomaterials. BC is a non-pathogenic bacteria-produced polysaccharide with a 3D nanofibrous structure, chemically identical to plant cellulose, but exhibiting greater purity and crystallinity. Bacterial cellulose possesses excellent physicochemical and mechanical properties, adequate capacity to absorb a large quantity of water, non-toxicity, chemical inertness, biocompatibility, biodegradability, proper capacity to form films and to stabilize emulsions, high porosity, and a large surface area. Due to its suitable characteristics, this ecological material can combine with multiple polymers and diverse bioactive agents to develop new materials and composites. Bacterial cellulose alone, and with its mixtures, exhibits numerous applications, including in the food and electronic industries and in the biotechnological and biomedical areas (such as in wound dressing, tissue engineering, dental implants, drug delivery systems, and cell culture). This review presents an overview of the main properties and uses of bacterial cellulose and the latest promising future applications, such as in biological diagnosis, biosensors, personalized regenerative medicine, and nerve and ocular tissue engineering.

## 1. Introduction

Over the last decades, due to the advancement of technology (artificial intelligence or robotics) [1], wide novel, multifunctional, and biomimetic biomaterials (natural, modified natural, or synthetic) have been developed [2] with enhanced properties and applications [3] suitable for use in areas from the food industry to regenerative medicine and bioprinting [4]. These biomaterials can be successfully substitute for the traditional materials [5]. The term ‘biomaterial’ refers to an eco-friendly material, which is based on sustainable resources (agricultural raw materials, fossil, and electronic reserves) [6]. The extensive development of novel chemical and physical methods furnishes new opportunities for the scientific community to study and research particular elements to design effective and safer materials to ensure the regeneration of impaired skin [7]. The researchers have a tremendous interest in the entire groups of biomolecules (monomers, oligomers, and macromolecules), such as carbohydrates, amino acids, proteins, nucleotides, nucleic acids, and lipids [8,9]. The common classification of biomaterials consists of four distinct classes: polymers, composites, ceramics, and metals [7]. For the biomedical and pharmaceutical fields (tissue engineering, wound dressings, bioimaging, drug delivery systems, implants, biosensors, biomedical diagnoses, and treatment of various conditions) [10,11,12], the new projected materials should display similar biological and structural characteristics, such as the indigenous extracellular matrix [13]. The novel biomaterials should have the capacity to sustain their structural stability to assure cellular proliferation and the development of new skin tissues [14]. Fundamentally, these biomaterials show biocompatibility, biodegradability, non-immunogenicity, non-cytotoxicity, biological inertness, histoconductivity, histoinductivity [15], bioactivity, optimum physicochemical, and mechanical properties in order to be used in the biomedical area. Their main purpose is to restore or to replace the skin tissue functions to increase the patient’s quality of life [16]. An essential feature of these newly designed biomaterials consists in their biosynthesis using harmless and safe technologies for the environment, known as ‘green’ methods, that considerably reduce the negative consequences of pollution on the climate and the human body [17].

Among these newly tailored biomaterials, a central place is occupied by bacterial cellulose (BC), an ecological polysaccharide broadly studied for multiple applications due to its excellent physicochemical and biological properties [18]. Bacterial cellulose is derived from cellulose, a natural polymer ubiquitously found in our surroundings, known as ‘the most plentiful biopolymer’ on Earth [19]. It is synthesized by all classes of plants, ranging from fungi, algae, and bacteria to cotton, wood, and hemp. Due to its abundant, renewable, degradable, and recyclable character, cellulose has gained attention as a sustainable material [20]. Cellulose is a hydrophilic polysaccharide consisting of linear macromolecular chains of 1–4 linked *β*-D-glucopyranosyl units forming linear chains in the cell wall [21]. Its high strength, stiffness, crystallinity, and durability are due to saccharide chains held together by Van der Waals forces and hydrogen bonds. Its overall reactivity is results from the presence of hydroxyl groups and their distribution [22,23,24]. Since the structural and chemical particularities of cellulose are covered in many papers, it will not be detailed in the current article.

Among the characteristics of bacterial cellulose are high biocompatibility, biodegradability, non-toxicity, viscoelasticity, flexibility, chemical stability [25], adequate hydrogel traits [26], unidirectional polarity, and fluctuating density [27]. In comparison with the cellulose produced by plants, bacterial cellulose has a higher crystallinity [28], purity, tensile strength [29], value of degree of polymerization, and Young’s modulus [30]. It has a hydrophilic porous structure that allows it to retain a large quantity of water (>90%). BC has a large applicability in fields ranging from electronics, paper, and food [31,32] to applications in the biomedical industry (tissue engineering, bone and cartilage reconstruction, implants, wound dressings, cornea restoration, artificial blood vessels, orthodontic treatment, drug delivery devices, antibacterial products, biosensors, biological diagnoses, regenerative medicine), the pharmaceutical field, veterinary medicine, the leather industry, and pollution control [33,34,35].

In this review, we will further present the main aspects concerning bacterial cellulose biosynthesis and its properties and applications with emphasis on its biomedical uses, such as in dressing materials and artificial scaffolds [36]. We will discuss various combinations of BC and different biopolymers (natural and synthetic) with several bioactive agents (metals, inorganic substances, plants extract, or drugs) to develop new materials and composites with a large applicability in the biomedical and biotechnological domains [37].

## 2. Bacterial Cellulose—Pioneer for Continuously Developing Macromolecules

### 2.1. State of the Art

Bacterial cellulose (BC), also known as bacterial nanocellulose (BNC) due to its nanostructured network [38], represents a particular biopolymer [39] produced by certain bacterial strains through fermentation processes; it is lignin and hemicellulose free [40]. It is also called microbial cellulose [41]. First reported by Brown in 1988, this natural polymer resembles plant and wood-derived cellulose, exhibiting the highest purity compared to the latter. Structurally, bacterial cellulose mainly consists of nanofibrillar polysaccharides, which have a diameter between 20 and 100 nm. Thus, bacterial nanocellulose is much thinner than the cellulose extracted from plants [38]. Bacteria produce extracellular cellulose fibers, in static or even dynamic conditions, with different yields, depending on the species and also on the culture media substrate. *Komagataeibacter xylinus* (also known as *Gluconacetobacter xylinus* or *Acetobacter xylinum*) [39], a strictly aerobic Gram-negative bacterium, is known as the strongest cellulose producer, along with other species: *Komagataeibacter medellinensis*, *Komagataeibacter* *hanseii*, *Komagataeibacter oboediens*, *Komagataeibacter rhaeticus*, and *Komagataeibacter pomaceti*, classified as safe bacteria (GRAS). Other bacteria known to produce BC, *Azotobacter*, *Escherichia*, *Pseudomonas*, *Rhizobium*, *Salmonella*, *Agrobacterium*, *Klebsiella*, and *Sarcina ventriculi*, are reported, along with recently discovered *Lactobacillus hilgardii* [42,43,44].

### 2.2. Biosynthesis

Bacterial cellulose is synthesized by oxidative fermentation in a synthetic and non-synthetic medium. The earlier-presented non-photosynthetic microorganism *Komagataeibacter xylinus* is fermented at pH = 3–7 at a temperature of 25–30 °C using a saccharide as a carbon source and producing a large quantity of cellulose microfibrils [45,46,47].

**Figure 1:**
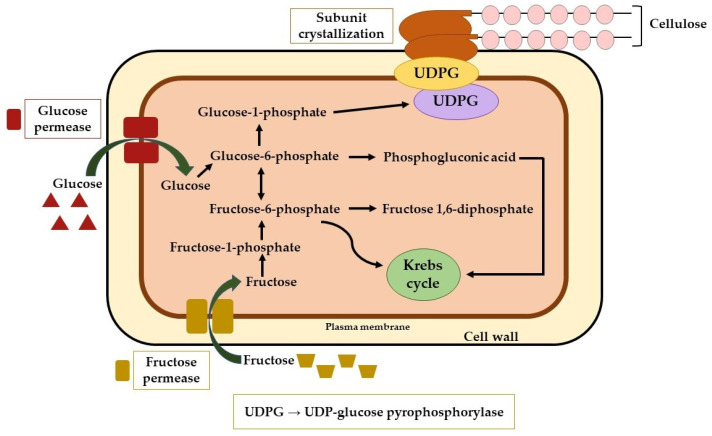
The microscopic cytoplasm synthesis of bacterial cellulose.

Bacterial cellulose originates in the bacterial cytoplasm and is carried out in the membrane of the microorganisms. With glucose as the substrate, the cytoplasm becomes the host of a reaction cycle: phosphorylation–glucokinase, isomerization–phosphoglucomutase, and uridine diphosphate UDP–glucose (UDPG) are produced [48]. The bacterial cellulose cytoplasm synthesis stage at a cellular (microscopic) level is illustrated in Figure 1.

The next stage occurs in the membrane where cellulose synthase operates. This enzyme is the main determinant of the cellulose type, along with the starting substrate. Thus, the nucleotide-activated glucose chains are extruded through pores, resulting in d-glucose units interconnected through *β*-1,4-glycosidic bonds. More bonds are formed between these after leaving the ‘mother-organism,’ resulting in longer chains which are inter- and intra-linked by hydrogen bonds measuring approximately 25 nm in width and up to 9 µm in length. Further, the linkage between these structures determines the formation of ribbon-shaped fibrillar formations (<100 nm in width). During culture, bacterial cellulose morphology transforms from the ‘floccus’ to the ‘pellicle’ phase, translated in a 3D-network to resemble a pellicle at the surface of the culture media [49,50,51,52].

After fermentation occurs in the culture media and bacterial cellulose is obtained, a purification process is mandatory since the product is not of high purity and may contain culture media residues (for example, lignin and hemicellulose), unwanted cells, or side products [53,54]. A standard, but expensive, purification procedure requires the following steps: harvesting the bacterial cellulose pellicles from the culture vessel, washing them in distilled water to remove any residual medium, treating them with NaOH/KOH/Na_2_CO_3_ at 100 °C for 15–20 min to kill the microorganisms, filtering them using an aspirator, and finally neutralizing the filtrate using distilled water. A drying method is further applied before obtaining the final product [55,56]. Another method of purification also uses an alkaline medium, NaOH or K_2_CO_3_ at 80 °C for 60 min; the procedure is performed twice to eliminate all the bacteria [57]. The purification of bacterial cellulose is of crucial importance when its cultivation targets are the fabrication of wound-healing materials, especially cartilage implants [58]. Researchers point out that supercritical CO_2_ processing and treatment with carbonic acid under high pressure are sufficient purification procedures for microbial cellulose that will be used in biomedical applications. Recent experiments indicate that treatment in biphasic systems is more effective in terms of maintaining the main structure of the cellulose network [59].

One of the aforementioned factors impacting bacterial cellulose synthesis is the culture media. In other words, the carbon and nitrogen sources are the most important. The culture substrates involved is the main reason why obtaining bacterial cellulose is an expensive process. Following eco-friendly fabrication protocols, wastewater from distilleries was reported as an efficient culture media for bacterial cellulose. The method is advantageous because it produces quality bacterial cellulose and diminishes waste disposal by transforming the wastewater into a cheap fermentation promoter [60]. Other parameters such as pH, culture type (static/dynamic, surfaced/submerged), shear forces, and oxygenating rates of the support also influence the synthesis process.

In terms of bacterial cellulose networks, the literature data include different patterns, depending on the type of culture conditions. For example, solid-state cultures of *Gluconacetobacter xylinus* have been reported to produce bacterial cellulose with a honeycomb geometry, following a polyurethane support pattern. Also, the use of support enhances biomass recovery [61]. Other honeycomb-like matrices were obtained from bacterial cellulose and gelatin, exhibiting large surface areas and uniform pore arrangement. Ampicillin was added, resulting in a retarded release sponge with potential antibacterial applications [62].

### 2.3. Properties

**Figure 2:**
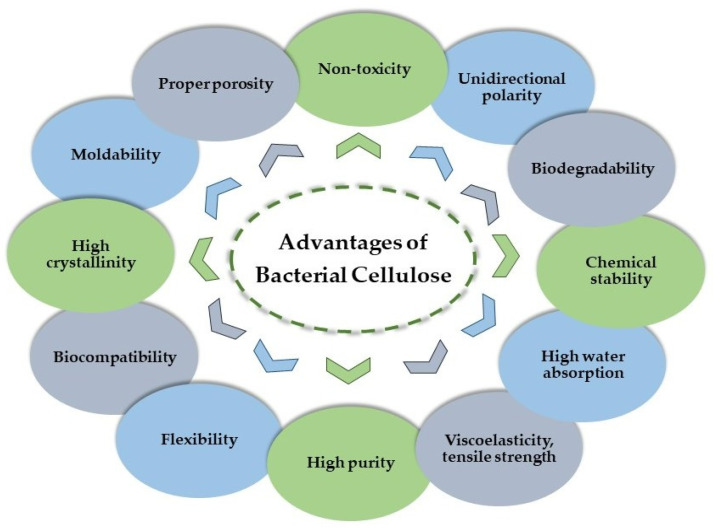
The advantages of bacterial cellulose.

As expected, the properties of microbial cellulose reside in its structure. It consists of bundles of cellulose nano-fibrils (2–4 nm), which become ribbon-like structures of around 100 µm in diameter and 100 µm in length [63,64,65,66]. Due to this particular nanostructure, bacterial cellulose has the capacity to retain its dry weight inside water; this fact gives this biopolymer superior elasticity, flexibility, and resistance in humid conditions; therefore, bacterial nanocellulose represents an excellent natural resource for designing new bandages for the accelerated healing of lesions [38]. It exhibits an ultrafine web structure which is very difficult to disperse in water [67]. The crystallographic structure is similar to that of cellulose type Iα [43,68]. Compared to other biopolymers, bacterial cellulose exhibits a combination of unique properties due to its fibrillar structure and its light weight, its purity, and its macromolecular properties, including its polymerization degree of up to 8000 [69]; its other characteristics such as its hydrophilicity, crystallinity (up to 90%) [70], moldability, non-toxicity, and biodegradability also set it apart [71]. The pure cellulose nanofibers confer intrinsic high purity and strength without the need of further refining treatments [42,63,71,72]. Chemical modifications are also permitted due to vacant hydroxyl groups that are not engaged in hydrogen bonds [49]. Its unique optical, electrical, and mechanical properties with numerous improvement possibilities have attracted the attention of scientists in many fields [73]. Bacterial cellulose morphology can be changed as needed. The literature data report these biogenesis interventions: growing cellulose in a chitosan-modified culture media or a methylcellulose-carboxymethylcellulose-poly(vinyl alcohol)-modified media for its application in wound dressings or other medical applications [74,75]. The main advantages of bacterial cellulose are summarized in Figure 2.

### 2.4. Applications

Individually, bacterial cellulose has been an excellent starter for many applications due to its particular physicochemical and biological properties. The direction of its applicability points primarily towards the biomedical field [76]. These are a few examples of how bacterial cellulose can be included in innovative biomedical applications.

First of all, microbial cellulose is a suitable material for 3D-bioprinting. The literature data describe bacterial cellulose as a medical material used in bioprinting costal, auricle, and nasal cartilage, due to its special 3D-structured network and unique properties [77]. By increasing its cellulose content by 17%, modified bacterial cellulose showed similarities to human auricular cartilage one week after its implantation. High compatibility was also demonstrated, along with non-absorbable properties, as the implant was accepted by the surrounding soft tissues [78].

Other challenging therapies where BC may play a vital role involve oral implants and newly guided bone regeneration techniques. BC has proven to exhibit suitable characteristics in the role of a barrier membrane, a component of capital importance in implantology. BC enhances tissue and bone regeneration by separating them from other surrounding tissues [79]. Moreover, BC is an important candidate to replace collagen (cytotoxic) as a shielding membrane, since its rate of biodegradation can be reduced [80]. It also exhibits a promising potential as a root canal treatment material, with perspectives in replacing commercially available disposable paper points [81]. Experimental studies showed that potato starch added to a BC culture media produces an increased viscosity and fills many networks’ vacant spaces. Moreover, scaffolds are obtained after culturing muscle cells onto the surface. This pattern complies with the specifications of hollow organ reconstruction material [82].

BC also represents a feasible material for tissue engineering [83], a field that is currently focused on discovering materials and techniques to artificially mimic a suitable environment for stem cell culture [84]. It is well known that stem cells stand out because of their self-renewal characteristics emphasized by their ability to further differentiate into numerous cell types, depending on the specificity of the organism in question, with a high potential for multiple applications [85,86]. A research team demonstrated how bacterial cellulose can be exploited in this direction: a nanofibrous bacterial cellulose membrane is reported to have the ability to inhibit the differentiation of mouse embryonic stem cells. At the same time, the mouse embryonic fibroblast cultivation was improved, in comparison to the conventional culture media. The pluripotency of the cells was confirmed, along with their ability for quick manipulation, significantly enabling other handling maneuvers [87]. BC also has properties which make it adequate to serve as a scaffold for tissue engineering at the level of the cartilage. Chondrocyte proliferation studies developed using BC supports have been carried out. Bacterial cellulose loaded with bone marrow mesenchymal stem cells also represents an innovative resource for developing scaffolds and quality testing techniques for bone reconstruction materials. A study using horse stem cells proved BC’s cell adhesive and life supporting platform properties [88]. Due to it being non-cytotoxic, BC may function as a durable scaffold in the slow-healing processes [89]. Outstanding studies were carried out to improve and discover new methods for diagnosing neurodegenerative diseases. Results showed that neuroblastoma cells (SH-SY5Y) attached and proliferated on a bacterial nanocellulosic 3D-scaffold, resulting in mature neurons. This special model was designed for the investigation of neurodegenerative disease mechanisms, paving the way for discovering new treatments for neurological conditions [90].

Another branch of the biomedical field where BC occupies a central place is in the development of wounds dressings for the treatment of lesions of different etiologies (burns, chronic skin ulcers, surgical incisions, and traumatic wounds) [91]. BC exhibits a high capacity to maintain an optimal moisture at the lesion site, to absorb wound exudates, to allow a good exchange of gases, to provide thermal isolation, and to prevent a strong adhesion to the skin tissue. BC-based wound dressings supply an excellent protection against contamination and infection, reducing the occurrence of local pain and inflammation. Therefore, all these advantages lead to an increase in skin restoration and re-epithelialization, accelerating the wound healing process [92].

Along with the biomedical applications of BC presented above, BC can also be a promising material for controlled drug delivery [93]. Thus, BC can be loaded with various drugs, such as benzalkonium chloride, tetracycline, ibuprofen, diclofenac, paracetamol, propranolol, lidocaine hydrochloride, caffeine, silver sulfadiazine, and amoxicillin [92,94].

The second direction of applicability for bacterial cellulose is in the pharmaceutical domain. The use of intelligent technology in the pharmaceutical industry represents the future in terms of delivering active moieties to specific sites of absorption and action. Among these, Pickering emulsions have gained interest in recent years due to their versatility and possible application in the pharmaceutical and cosmetic industry. Recent approaches to these disperse systems include liquid marbles as precursors [95] and cellulose nanofibers/nanocrystals as stabilizers. Investigations in this direction include the study of oil droplets trapped by cellulose nanocrystals. Further studies revealed that the Pickering emulsions can be stabilized using TEMPO-oxidized (2,2,6,6-tetramethyl piperidine oxide) bacterial cellulose nanofibers. The assessment of the quality parameters of these emulsions indicates stability and viscoelasticity, and therefore, great potential for its use in new drug delivery systems [96].

The third direction of applicability of bacterial cellulose is in the biotechnological industry [97]. The petrochemical-based industry flourished for many decades, but nowadays a migration towards a bio-based ‘green’ economy is unfolding. This implies that avoiding natural material exploitation comes first when developing quality bioproducts to be obtained according to modern world requirements. In recent decades, the researchers’ interest was focused on BC as a promising ecological biomaterial due to its eco-friendly production process. Furthermore, BC possesses adequate mechanoelectrical and electromechanical transduction characteristics [98]. Following this direction, biofibers are currently important candidates as reinforcements for numerous applications of polymer composites [99]. Due to its suitable electrical properties and its renewable capacity, BC has a high potential for use in the expansion of new biosensors, wearable electronics, biomedical and energy storage devices, electrodes, and supercapacitors [100]. Biosensors have a large applicability in tissue engineering and regenerative medicine. Among them are enzymes, receptors, and antibodies. These devices are broadly used in bioanalysis because they can verify the biological signals in real time, informing health care providers about the current health status of the patient, helping the medical team to discover the disease in time and to initiate the treatment [101].

## 3. Bacterial Cellulose Composites—Important Emerging Materials for Biomedical Design and Other Impacting Applications

It is well known that ‘composites’ represent an umbrella term, defining a class of compounds obtained by placing two or more types of materials together: one plays the role of the matrix and the other is the reinforcement. The final product meets high-quality standards when compared to the raw materials it is made of [102].

**Figure 3:**
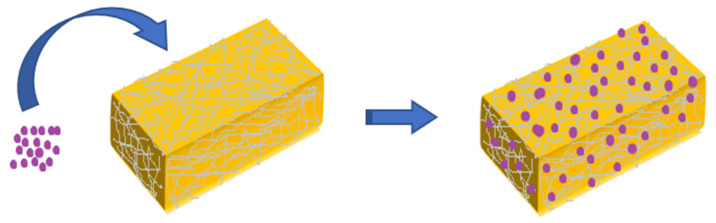
A schematic illustration of a bacterial cellulose composite.

Taking into account the aforementioned aspects and the fact that bacterial cellulose is a 3D-structured network, BC may function as a matrix to ‘trap’ other potential compounds [103]. Thus, microbial cellulose composites came to life, aiming to improve and eliminate some of the components’ disadvantages or to adapt a range of properties, depending on the desired final product [104]. The direct addition of various materials applied to BC results in a ‘combination product’ called BC composite, as illustrated in Figure 3.

Bacterial cellulose presents a high capacity to act as a host for some oxide nanoparticles (metals, carbon derivates, minerals). The many possibilities for using BC as a mesh for obtaining composites are becoming popular, and most of them involve other known polymers [105].

### 3.1. Biopolymers for Tailoring Bacterial Cellulose-Based Composites

The term ‘polymer’ is ubiquitous and defines a widespread class of materials used in many industrial areas. As many would say, we live in a so-called ‘polymer age,’ which is exhibited by several changes that a polymer can suffer, such as physical, chemical, thermal, or photochemical modifications [106]. The expanding role of these macromolecules in our lives is the reason why, during the last decades, scientists became interested in developing value-added products based on polymers. Currently, the main concern is obtaining biocompatible polymers via ecological and economical techniques. These biopolymers are environmentally friendly with low toxicity, making them suitable for multiple applications [107]. The waste disposal problem arising from industrial expansion is one of the main reasons why self-biodegradable materials became a necessity. Thus, in the 1980s, biodegradable plastics were created, originating from natural and synthetic polymers [108,109]. The newly designed materials exhibited the characteristic of self-biodegradation in the presence of living microorganisms which act in collaboration with chemical factors or enzymes [110].

Currently, worldwide efforts concern preference of biodegradable materials over non-biodegradable ones. The scope is to diminish pollution and uncontrolled waste. Polymers were once considered pollutant agents and most of them still maintain their ‘bad reputation’ [111]. As it is well known, a polymer is composed of repetitive similar/non-similar entities, called monomers, which connect through non-covalent bonds, generating the polymolecular entities [112]. A brief classification of the most widespread polymers includes cellulose, collagen, chitosan, and hyaluronic acid as natural polymers [113]; cellulose derivatives (sodium carboxymethylcellulose (NaCMC), hydroxyethylcellulose (HEC), hydroxypropylcellulose (HPC), hydroxypropylmethylcellulose (HPMC), methylcellulose (MC), and ethylcellulose (EC)) as modified natural polymers obtained by modifying the pure cellulose through the etherification reaction with alkyl groups [114]; and poly(vinyl alcohol) (PVA), polyvinylpyrrolidone (PVP), and Carbopol^®^ as synthetic polymers [115].

All biopolymers stand out because of their remarkable previously discovered applications, but also distinguish themselves by perspectives of these properties, which should be brought to light. Among them, this paper points out a few innovative applications. The focus will be placed on emerging materials in the biomedical field (tissue engineering, wound dressings, drug delivery systems, regenerative medicine) where biopolymers act as precursors [116]. Thus, these biomaterials present biological functions which allow the healing of any impaired organ or tissue of the human body [117].

Due to the excellent physicochemical and biological properties of all types of biopolymers (natural, modified natural polymers, and synthetic), they can combine with bacterial cellulose to develop new composites with results that surmount their drawbacks and extend their applications [102]. BC-based composites have a large applicability in the wound healing process as wound dressings, as well as in tissue engineering, implants (bone, cartilage, cornea, teeth), 3D-bioprinting, the treatment of cardiovascular and neurological diseases, drug delivery, biosensors, electronics, and biofuels [102,118], as will be described below.

Cellulose, one of the main natural polymers, may experience different structural changes through specific processes to attain better quality (to improve its water solubility) and to extend its applicability in multiple domains [119]. Among the methods applied, lyophilization was mentioned in the literature data as a method to increase the porosity of a cellulose matrix for favorable bone regeneration [120]. Mello et al. obtained lyophilized cellulose and assessed it as a wrapper for peripheral nerve injuries (sectioned sciatic nerves) in animal studies. It was demonstrated that lyophilized cellulose caused a moderate fibrous reaction when implanted in peripheral nerve lesions with loss of substance. It proved to be effective as protection in those lesions in the presence of an inserted neural graft. Axons regrowth was reported, along with motor response after a period of recovery [121].

Other applications that indicate the use of cellulose-based materials pertain to the restorative medicine field [122]. One such material is oxidized regenerated cellulose (ORC), a natural biopolymer with carboxyl groups, which results through the chemical oxidation of pure cellulose. This material possesses several optimal characteristics (non-toxicity, biodegradability, antibacterial activity, and biocompatibility); thus, ORC has multiple medical uses, but it is principally a hemostatic agent [123]. A retrospective study on patients that underwent skin graft reconstruction treatments used ORC and a collagen-based composite loaded with silver. Results indicated a reduction in pain medication usage during healing, along with a decreased necessity for dressing replacement [124].

Collagen is a natural polymer known as the major constitutional protein found in human tissues. It makes up the extracellular matrix of connective tissues. Due to its high biocompatibility, it has been used in various biological applications, including as a wound dressing material, part of drug delivery systems, and materials-based scaffolds for tissue engineering [125]. Due to its suitable properties, collagen can be combined with other polymers, for example, with dextran in a spongious matrix loaded with flufenamic acid. Release profiles were obtained from animal testing: gradual delivery of the anti-inflammatory moiety accelerated the wound healing process and also enhanced re-epithelialization. The aim of designing these matrices was to reduce burn lesion progression, pain, and inflammation. The exact mechanism involved in tissue restoration through these particular systems will require further investigations [126].

Along with the above association (collagen and dextran), collagen can be also combined with BC for possible applications in the biomedical field. Different forms of collagen (hydrolyzed, solution, gel) embedded onto BC sheets increased their quality in terms of thermal stability and improved mechanical properties over plain lyophilized BC [127]. New research is constantly being carried out to guide collagen and other natural polymers towards inclusion in high-quality wound dressings. Rheological parameters in correlation with biological behavior and structure were studied for a spongious collagen-dextran-zinc oxide (50%) composite. Important perspectives for skin regeneration and antibacterial properties were indicated [128].

Moreover, collagen, the main component of bone tissue, is included in numerous composites (with BC), which are proposed as regeneration processes enhancers [77,129]. BC-collagen scaffolds impregnated with human umbilical cord blood-derived mesenchymal stem cells successfully functioned in osteogenic differentiation. Subcutaneous transplantation of these scaffolds enabled prolific neovascularization in early bone regeneration [130]. Vascular endothelial growth factor was added to BC scaffolds in studies carried out on femoral fractures in mice and rabbits, proving promotor properties in vascularization, ossification, and maturation of newly developed bones [130,131]. Playing a role similar that of plain BC acting as a separator between tissues in oral implants recovery, BC-collagen is applied as a carrier material for the osteogenic growth peptide with the goal of conserving bone defect space during the healing process, as well as enabling hyperplasia [132]. Moraes et al. developed a hydrogel dressing based on BC and collagen and compared it with a commercial product regarding their effect on the healing of rat dorsum. In vivo studies showed better skin healing using the newly designed hydrogel on day 7 after surgery [133].

Apart from physical contact therapies in wound healing and scaffolds, research groups also studied drug absorption and release mechanisms through porous microspheres based on collagen and bacterial cellulose. Absorption equilibriums analysis indicates promising applications for this system in future active moiety delivery systems for wounds [134].

Along with the natural polymers mentioned above, chitosan is also widespread in nature, second only to cellulose. Its origin is in the natural chitin. Chitosan exhibits many excellent properties, such as biocompatibility, non-toxicity, biodegradability, particular solubility, along with antimicrobial [135,136], antioxidant [137], antiviral, and antifungal effects [138]. Chitosan is well known as an excipient for drop-type ophthalmic products [139], and also for complex entities like liposomes [140], microemulsions [141], hydrogels [142], and implants [143]. Due to its excellent characteristics, chitosan can be used as a scaffold alone or in association with other polymers to develop new materials with promising biomedical uses. For example, chitosan in a gel form made was included as part of a drug delivery system destined for periodontal diseases treatment through intra-pocket drug release. As such, one proposed approach included chitosan-based formulations containing a chemotherapeutic agent (metronidazole benzoate) and an antibiotic (tetracycline hydrochloride). The optimum chitosan concentration was established through kinetic profile analysis. Thus, the gel with 3% *w/w* chitosan represents an excellent local treatment for periodontitis [144].

Moreover, ophthalmic pharmaceutical forms represent a challenge in terms of formulation and organ specificity. Apart from the active ingredients, auxiliary components are the ones entitled to encompass these boundaries. Chitosan won an important position in this direction due to its biodegradability, bioavailability, and permeation enhancement ability. Moreover, its antibacterial and antifungal properties, along with its intrinsically adhesive nature, promote chitosan’s inclusion in modern ophthalmic drug delivery systems. Since most in situ ophthalmic chitosan gels commonly deliver only one active substance, future investigations will try to incorporate more active ingredients, paving the way to attaining a local synergistic action [145,146,147].

In the meantime, chitosan can also be combined with BC. Thus, it was included in BC-based composites with various applications. The literature data included a BC-chitosan film that was compared to plain BC and other hydrocolloid films (Tegaderm^®^). It was demonstrated that BC did not dehydrate wounds but maintained a suitable moist healing environment with good permeability. The BC-chitosan film enabled skin regeneration and provided a better wound-healing effect [148]. Bacterial cellulose and chitosan, along with ciprofloxacin, were successfully integrated into a patch with dual antibacterial properties [149]. The mixture of BC, chitosan, and carboxymethylcellulose led to an antimicrobial film with a higher tensile strength and water vapor transmission rate [150].

In the category of the modified natural polymers, a central place is occupied by the cellulose derivatives (CMC, HPMC, MC, HEC, HPC, EC) that are of great interest, mainly in the food industry [151]. Nowadays, every industry is trying hard to maintain a standard of low waste, suitable economy, and high quality. The food industry concentrated its efforts on the development of cellulose derivatives as proper materials for food packaging and freshness maintenance due to their antimicrobial effect [152]. In this direction, many tests were performed, such as the scanning electron microscopy (SEM) analysis on gluten networks, showing that CMC is suitable to be used as a flour dough rheology regulator [153], whereas HPMC functions as a texture enhancer for whipped cream [154]. Following the idea of improving food preserving methods, methylcellulose-coated eggs exhibited promising shelf-life freshness when compared to eggs with uncoated shells [155]. Another functional food interface was developed targeting antioxidant activity using methylcellulose plasticized films, showing efficacy in preserving tocopherol content in walnut oil [156].

CMC also qualifies as an important polymer for use in the biomedical field due to its biocompatibility with human skin, its biodegradability, and its non-toxicity. Its advantages also include its high-water uptake, which is very important in ensuring a favorable environment for re-epithelialization [157]. NaCMC has a great ability to combine with other polymers to generate new composites showing suitable applicability in the biomedical field. For example, to meet all of the qualitative requirements of wound healing materials, spongious matrices comprised of collagen-NaCMC-mefenamic acid were developed and tested. Ninety-five percent of the anti-inflammatory agent was released. Along with favorable degradation and swelling ability, these hybrid matrices were proposed for further in vivo and in vitro testing [125]. Moreover, this combination of collagen and NaCMC and the same anti-inflammatory drug, mefenamic acid, was comparatively tested on rats in terms of its burn healing efficacy. Results indicated valuable effects in the hemostasis and inflammation stages, accelerating wound healing through the reduction of pain and minimal scar formation [158]. Local treatment for burns and soft-tissue injuries also included a multiparticulate system based on a collagen-dextran matrix embedded with flufenamic acid. Apart from the polymeric matrix, it consists of microcapsules based on gelatin-NaCMC-alginate with an embedded anti-inflammatory drug. This model was proposed as a promising design for future similar applications [159]. CMC can also be blended with BC. Thus, Pavaloiu et al. designed a new composite hydrogel loaded with ibuprofen sodium to study its drug release and swelling characteristics. It was found that the mechanism of swelling is controlled by pseudo-Fickian diffusion [160]. Juncu et al. formulated composite films based on NaCMC, BC, and ibuprofen sodium. The swelling behavior was studied using non-linear diffusion. The main results showed that the drug delivery depends on the content of BC; thus, the increase in the concentration of BC led to a decrease in the ibuprofen release rate [161].

Of special interest is the association between sodium alginate and BC. Cartilage restructuring properties were discovered in the BC-alginates double-layered composites inoculated with human nasal septal chondrocytes. In vitro culture revealed the stents’ potential as a model in treating severe auricle defects, as new healthy cartilage was formed through the porous layer of the biocomposite [162]. Porous sponges were obtained by combining BC and sodium alginate and cross-linking this with CaCl_2_ solutions. The sponge is conceived as a tear resistant, daily removed dressing for covering oral cavity surgical wounds [163]. The BC-alginate-chitosan-copper composites were confirmed to be suitable for use as biocompatible wound dressings due to their antibacterial activity against *Escherichia coli* and Methicillin-resistant *Staphylococcus aureus* [164]. Alginates were included, along with bacterial cellulose, in composite films loaded with silver sulfadiazine, proving cytotoxicity and an extended antimicrobial spectrum (*Escherichia coli*, *Staphylococcus aureus*, and *Candida albicans*) [165]. Kim et al. designed a BC and alginate-based nanocomposite, with normal spherical shapes and size, that showed high biocompatibility, biodegradability, optimal capacity to absorb water, higher crystallinity, and greater surface area. All these suitable properties enhanced the field of use for this nanocomposite, with promising applications in the biomedical, pharmaceutical, and biocatalytic industries [166].

Included in the category of synthetic polymers are poly(vinyl alcohol) (PVA), polyvinylpyrrolidone (polyvidone) (PVP), polyacrylic acid (Carbopol^®^), poly(ethylene glycol) (PEG), polyacrylamide, and polyurethanes, which are hydrophilic substances with swelling properties in the presence of biological fluids, thus generating hydrogels [113,115]. Two of them, PVA and PVP, are widely used in ophthalmic formulations (contact lenses and artificial cornea) [167,168] due to their optimal biocompatibility, biodegradability, capacity to form films, transparency, proper viscosity, excellent ocular mucosa penetration, and eye contact [169,170,171]. Moreover, PVA exhibits an appropriate capacity to blend with other polymers such as collagen to develop a novel matrix for tissue regeneration. The matrix was embedded with indomethacin as an anti-inflammatory molecule, resulting in a stable biohybrid. Kinetic parameters indicated an initial burst release of indomethacin, followed by slow drug delivery. Thus, the proposed spongious delivery systems presented promising results for replacing classical formulations used for tissue recovery purposes [172]. Moreover, PVA can also be combined with BC, creating a new composite that controls drug-delivery rates for anti-inflammatory substances. The system included poly(vinyl alcohol), chitosan, and bacterial cellulose; experimental results proved their possible use as biocarriers [173]. A new nanocomposite was obtained by blending BC with PVA and magnetite nanoparticles, which exhibited proper characteristics for use in the development of smart electronic devices [174].

**Figure 4:**
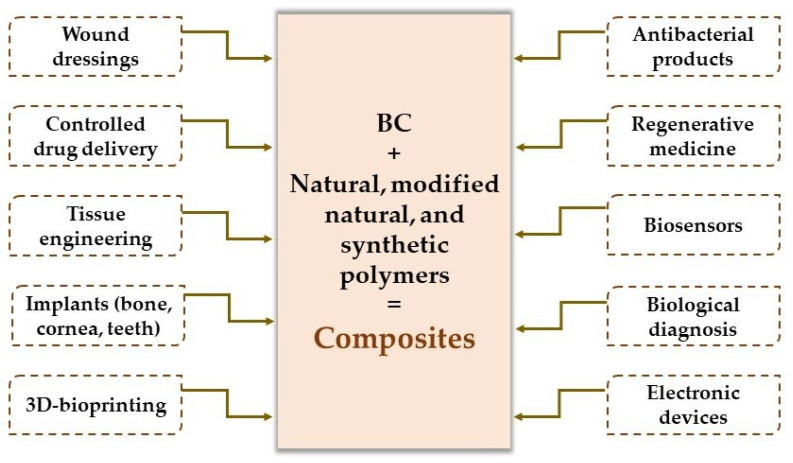
The major applications of bacterial cellulose-based composites.

The major applications of bacterial cellulose-based composites are highlighted in Figure 4.

### 3.2. Applications of Bacterial Cellulose-Based Composites

Bacterial cellulose is considered an efficient substance carrier. Due to its architecture, which resembles a porous network, it acts as an intermediary between the wound and an antimicrobial agent embedded in its structure. It is also a physical barrier against infectious agents. Many composites were created starting with these structural advantages. One example is the BC-NBG (nano-bioactive glass) composite which proved the synergistic antibacterial action of the two components. Laboratory tests showed antibacterial properties against *Escherichia coli*, *Salmonella typhymurium*, *Pseudomonas aeruginosa*, *Klebsiella pneumonia*, *Bacillus subtillis* [175]. It is also important to emphasize that the field of biomedicine has much to gain from attributes of bacterial cellulose, including high compatibility with the human organism and cost-effectiveness. Antibacterial composites include, among others: MH-BC (from mulberry leaves fermented in an acid hydrolysate fermentation medium) with proven perspectives in regenerative medicine [176].

Experimental data also showed that thymol-enriched bacterial cellulose has in vitro antibacterial activity against infectious bacteria, with prevalent potential for use in burned skin therapies. Cell viability and fibroblast proliferation were analyzed and the results showed an increase in the protection and coverage of damaged and recovering tissues. In vivo results indicated that wound closure and re-epithelization were not only enabled, but also accelerated [177]. Other research data revealed that biocomposites containing BC and *Bacillus subtilis* were reported as efficient and promising wound dressings, enabling full-thickness wound healing [178].

Bacterial cellulose and its composites also have effective applications in reconstructive medicine. The literature data indicated that accelerative wound healing processes and urinary reconstruction using angiogenesis promoters were achieved by using a combination of bacterial cellulose and urine-derived stem cells [179]. The potential of this association is noteworthy because of its telomerase activity, revealing the markers that are found on the surface of mesenchymal stem cells. Advantages compared to traditional wound dressings include an absence of secondary damage to the tissue and the absence of exudate accumulation. Bacterial infections are prevented due to bacterial cellulose being a skin substitute with high water-retaining capability [180].

Bacterial cellulose-based biomaterials used for wound healing are of major importance, particularly for the treatment of open injuries, severe burns, basal carcinoma, dermal abrasions, and chronic ulcers. Extensive tissue destruction or even severe infections may be triggered if wounds are not treated properly. Numerous studies introduced bacterial cellulose as an excellent dressing material. Many such products are already on the market: Biofill^®^, XCell^®^, Bioprocess^®^, Nanoderm^®^ [181], and also Cellumed^®^ (veterinary use) [182]. The literature data include reports of patients with second-degree wounds who exhibit faster healing when using BC-derived dressings compared to conventional products [183]. Wound dressing moisture balance is maintained, whilst skin is allowed to breathe and pain is reduced. Moreover, recent studies carried out on animals proved that wound dressing materials containing BC reduced the inflammatory response and improved wound healing and regeneration [184]. Other investigations revealed that the BC wound dressing materials exhibited superior covering properties for all facial contours and increased the cohesion levels on the mouth and nose regions, compared to other dressings; they also promoted a high moisture level in the wound, pain reduction, re-epithelialization acceleration, and a reduction in the appearance of scars [184,185,186]. Moreover, histopathological experiments proved that thick-BC wound dressings induced superior capillary formation, tissue regeneration, and cell proliferation compared to thin ones [187]. Clinical applications of BC-based wound dressing materials have the potential to replace classical gauze materials, as shown by experiments carried out on rat models [188].

As presented previously, a wound, and especially a burn infection, is an important aspect to consider during therapy because of its many triggered limitations. Pathogenic microorganism adhesion and further proliferation in the wound should be reduced as much as possible. BC-based dressings comply with these demands, the only difference being that no antibacterial protection is offered unless special treatments using organic (Ag, CuO, ZnO) or inorganic (lysine) agents are applied to the microbial cellulose fibers [188]. Bacteria are killed by impairing their main metabolic processes (respiration, nutrition) or by changing their cell wall structure and its normal function [189,190,191].

BC composites have been proven to make important contributions in the wound healing process, with previously demonstrated beneficial effects on hemostasis, inflammation, proliferation and remodeling phases of injury recovering. Even so, controversial aspects remain to be clarified, as many tissue healing mechanisms, especially scar formation and full recovery, are discordant [192,193].

Along with reconstructive medicine where wound healing processes benefit from bacterial cellulose composites, tissue engineering represents another field in which BC gains terrain compared to other materials due to its structured and porous 3D-network, biocompatibility, biodegradability, mechanical properties, and high power to retain large amounts of biological fluids [194]. Tissue engineering represents a new and demanding field of scientific exploration; it is an extensive multidisciplinary domain because it requires information from biology, chemistry, medicine, physics, and especially from engineering. The main purpose of tissue engineering is to expand several biological substitutes that can contribute to the anatomical and functional restoration, reconstruction, and increase in any human body tissue [195]. A biomaterial offers support and proper growth conditions in collaboration with other specific factors, promoting regeneration itself. Thus, the scaffold’s performance depends on its biocompatibility in terms of cellular adhesion and surface development, making the biopolymer responsible for cellular behavior (adherence, proliferation, migration) [196].

The ideal material destined for bone tissue engineering should exhibit certain properties: mechanical characteristics analogous to bone tissues, the ability to support the proliferation and differentiation of cells, the tendency to establish the deposition of the extracellular matrix [197], biocompatibility to support cellular interactions and tissue growth, biodegradability, absorbability, and last but not least, innocuity. Cumulative properties such as crystallinity and purity, in comparison to commonly used materials, promote bacterial cellulose as a superior qualitative medical material [198]. BC represents an ideal biopolymer that has a high capacity to simulate natural collagen due to its excellent properties mentioned above. Therefore, it is a remarkable candidate for use in bone restoration. Such an example is the mixture between BC and hydroxyapatite, a natural polymer that participates in the process of bone ossification. A new biomineralized BC scaffold has been designed with CMC as an activator of the BC surface; this novel formulation is a promising bone scaffolding material that requires further investigation [199].

Studies reported numerous BC-hydroxyapatite composites obtained through different technological processes as having various stoichiometric values, depending on the location and tissue for which they are intended. Bacterial cellulose was included, along with Fe_3_O_4_ and hydroxyapatite, in the scaffolds. Properties of the composite resemble human trabecular and cancellous bones. Further, in vivo investigations regarding this material’s osteogenic properties are needed for the scaffold to gain terrain against other classical prosthetic materials frequently used in dentistry [200].

Classical polymeric scaffolds do not retain high strength stability over time. To overcome this disadvantage, many techniques are still developing, mostly to obtain quality scaffolds for bone- and cartilage-recovering therapies. Polysaccharide scaffolds are mentioned in the literature as being compliant with physiological conditions, showing superior osteoblast adhesion and progressive bone mineralization compared to other control scaffolds (poly(lactic acid-glycolic acid)) [201]. Studies on nanocellulose scaffolds with collagen showed a greater adhesion and phenotype maintenance of cultured human osteoblasts, reflected by increased levels of alkaline phosphatase and mineral deposition compared to the control polyester micro-nano structured scaffolds of identical pore properties. These scaffolds are competitors for other polyester-based scaffolds used in bone restoration [202].

As per the case of bone tissue grafting, cartilage surgical repair also consists of similar procedures: the autografting of chondrocytes or osteochondral plugs. BC and its composites have been tested as cartilage replacement materials. One example includes the BC-poly(vinyl alcohol) composite, which exhibits the potential for use in the orthopaedical field as cartilage or intervertebral disc-replacement material [193].

Nowadays, materials with extreme wetting properties gain scientists’ attention because these materials present multiple ascending applications. Following the trend of developing biomimetic materials with special surface properties (for example superhydrophobic materials structurally resembling the lotus leaf [203,204]), bacterial cellulose became of interest to scientists working on hybrid materials. Thus, biocomposites with improved interfacial wettability were created by chemical cross-linking with oligopeptides, promoting tissue repair, which is of high importance in regenerative medicine [205]. Recent investigations pointed out the advantages of using bacterial cellulose in tympanic grafts, which enhances the surgical procedure by improving the healing ability after the graft is accepted by the organism.

One of the most studied and yet not fully understood systems, the cardiovascular domain, also benefits from the emerging application of bacterial cellulose. Hypertension and other heart-related pathologies are mainly caused by clogged or pathologically destroyed blood vessels. Scientists have developed polyester or polytetrafluoroethylene artificial grafts. Their inconvenience includes thrombi formation and appropriate capillarity, which is hard to obtain [45]. Thanks to its biocompatibility, high porosity, incredible mechanical properties, strength, and elasticity, BC became very popular as a vascular graft component. BC embedded with graphene oxide nanosheets is another composite developed to replace even small-diameter vessels [206]. Bypass implants registered a notable quality improvement when the product BActerial SYnthesized Cellulose (BASYC) was developed. Among its advantages, there are a few that stand out: mechanical strength in a moist state, smoothness of the interior lumen, and increased moisture preservation. Animal testing proved its success in the replacement of blood vessels using this material [45,207]. Other experiments regarding hemodynamics and physiological phenomena at the implantation site were carried out and these underlined the efficacy of the BC biosynthetic blood vessel precursors [186,208].

Even though BC and its composites proved to possess superior qualitative properties compared to conventional materials when referring to biomedical applications, scientists are still searching for new strategies to develop better composites. Recently, Gengiflex^®^ (based on BC) and Gore-Tex^®^ (based on polytetrafluoroethylene) membranes were tested for applicability in the dental field. Both membranes stimulated the bone expansion. After three months, the efficacy of the two products was compared. Thus, Gore-Tex^®^ showed higher efficacy than Gengiflex,^®^ and it is a promising candidate for healing osseous deficiencies [186].

As was previously stated, industrial fields gravitate towards quality materials and rely on obtaining them by combining well-known and studied materials. Following this flow, new composites were designed using bacterial cellulose. These products allow quality improvement in terms of mechanical, optical, and water absorption properties over plain bacterial cellulose [209,210].

Along with the applications of many of the newly designed composites highlighted above, a structured presentation of other bacterial cellulose composites, combinations of bacterial cellulose and various bioactive agents, and their biomedical applications are illustrated in Table 1. The proposed classification of these biomaterials and their prospective uses in the medical field were designed depending on tissue type and organ class. The table includes composites ranging from simple to complex structures, along with a wider range in terms of applications.

Along with all the aforementioned applications of BC, this biopolymer is also a promising biomaterial for biological diagnosis. To this end, Qin et al. developed a ‘living membrane’ system, which comprises BC and *Escherichia coli* bacterial strains, whose main purpose was to identify biologically triggered molecules [250]. Moreover, BC has a great potential for applicability in personalized regenerative medicine. A double network, biphasic Janus BC-conducting polymer composite hydrogel showed a biocompatible and electroactive behavior allowing the growth, spread, and migration of normal fibroblasts [250]. In recent years, the attention of the researchers has been focused on the development of BC-based biosensors used as monitoring devices. Marques et al. designed a BC-Ag nanocomposite-based biosensor intended to analyze 1-phenylalanine, 1-glutamine, and 1-histidine using Surface-enhanced Raman Scattering lamellas and 2 analytes (thiosalicylic acid and 2,2-dithiodipyridine) [251]. An enzymatic biosensor used for the amperometric determination of glucose has been developed using BC nanofibers and gold nanoparticles [247,252]. For the amperometric determination of the glucose oxidase reactions, Eisele et al. have created an external lamella in glucose biosensor with an extended range based on the use of BC and polyamide [253].

## 4. Conclusions and Future Perspectives

This present review has focused on the exposure of the synthesis, fundamental properties of bacterial cellulose, and its multiple applications in diverse domains, from food packaging to biotechnological, biomedical, and pharmaceutical industries. Bacterial cellulose is a valuable polysaccharide synthesized by a wide range of non-pathogenic bacteria under special culture conditions. This fascinating biopolymer possesses particular physicochemical, mechanical, and biological properties, such as eco-friendliness, biocompatibility, biodegradability, non-toxicity, a 3D-porous structure, optimal viscoelasticity, and tensile strength, an adequate ability to retain a large amount of water, moldability, along with higher crystallinity and purity than pure cellulose. It has been shown that bacterial cellulose can manifest a therapeutic effect on different anatomical parts of the human body, alone or in combination with several biopolymers and bioactive agents. Consequently, bacterial cellulose can act as an excellent medical material for the development of new skin lesion and dental dressings, drug delivery devices, oral implants, bone restoration or replacement products, local chemotherapy treatments, and cardiovascular interventional therapies. Presently, the attention of the researchers is centered on other captivating biomedical applications of bacterial cellulose, such as the development of biosensors, biological diagnoses, contact lenses, and nerve and ophthalmic tissue engineering. Besides all of the engaging uses illustrated above, bacterial cellulose is a promising material with high relevance for future use in the food, paper, and textile industries; acoustic membranes, supercapacitors, optical, stimuli-responsive, and catalytic materials; energy storage, oil refining, pollution control; and aerogels (reusable polymer networks that trap and dispense metal nanoparticles (Cu, Ni) used as catalysts in the electronic fields, due to their optimized properties).

## Figures and Tables

**Table 1:** Biomedical applications of bacterial cellulose.

Anatomical Part	Tissue Type	Application	Composition	Qualitative Properties	References
Skin	Epithelial tissue (soft tissue)	Wound restorative therapy	BC-modified topography	Wound healing enhancement: collagen migration enabled at the wound site along with fibroblast infiltration	[211]
			BC-CuO membrane	Proper antimicrobial activity against *Escherichia coli* and *Staphylococcus aureus*. It may function as a prototype for other similar products exhibiting photocatalyst and antimicrobial characteristics	[212]
			TEMPO-oxidized BC-AgNPs	Antimicrobial activity with 12% Ag release rates (37 °C).	[213]
			BC-TiO_2_	Antibacterial activity against *Staphylococcus aureus* and *Escherichia coli* proven on mice	[214]
			BC-AgNPs nanocomposite	Antibacterial activity against *Escherichia coli*, *Staphylococcus aureus*, and *Pseudomonas aeruginosa* due to the release of Ag; inflammation reduction	[215,216,217]
			BC-ZnO nanocomposite	Antibacterial activity against *Staphylococcus aureus, Escherichia coli*, *Pseudomonas aeruginosa*, *Citrobacter freundii*	[213]
			BC-propolis extract	Anti-inflammatory, antibacterial activity, and antioxidant functions on diabetic wounds	[209,218]
			BC-phenolic acids membranes	Suitable anti-inflammatory and antioxidant effects; non-cytotoxicity	[219]
			Periodate oxidized BC-chloramphenicol	Antibacterial spectrum, biodegradable, and biocompatible	[220]
			BC-vaccarin	*De novo* formation, neovascularization of tissues made of collagen, and fibrous connective tissue	[221]
			BC-diethyldithiocarbamate	OH-slow releasing systems: parasitic-caused lesion size reduction, SOD inhibition	[222]
			BC-ε-poly-l-lysine nanocomposite	Extended antimicrobial spectrum	[223]
			BC-acrylic acid hydrogel	Promoter of complete healing of wounds: water absorption and retention with good mechanical properties.	[210,224]
			BC-poly-methyl methacrylate	Biodegradable bandages, which support wound healing	[225]
			BC-Octenidine-Poloxamer BC-CMC-Methotrexate	Ready to use topical drug delivery systems: controlled release of active substances, effective for infected wounds	[226,227]
			BC-acrylic acid-human keratocytes and dermal fibroblasts hydrogel	Same wound healing properties as plain BC and a prolific cell carrier	[224]
		Enzymatic degradative biomaterials for surgical sutures	BC nanocrystals-regenerated chitin fibers	Wound healing enhancer with adaptable degradation rate (chitin concentration), biodegradable, strong suture material	[228]
		Tissue restoration	BC-tuned porosity	Muscle cell growth enhanced due to pore diameter, but slight strength reduction	[82]
			BC membrane	Appropriate nanomorphological properties, optimal control of infection, capacity to retain moisture; adequate drug delivery system	[229]
			BC-PHEMA hydrogel matrices	Mesenchymal stem cells proliferation proven in rats	[230]
	Connective tissue (transdermal level)	Active ingredients for transdermal release	BC-chloro-aluminum phthalocyanine membrane	Skin cancer: delivery system for photodynamic therapy with adequate properties for topical administration	[231]
			BC-lidocaine/ibuprofen membrane	Possibility of drug bioavailability modulation-dermal administration of lidocaine and ibuprofen	[232,233]
		Dressing materials	Modified BC-chitosan	Abdominal hernia treatment-reduced chance of infections caused by the mesh, no irritation, no hypersensitivity at implant site	[234]
			BC-sericin-PHMB film	Healing acceleration: low inflammatory response, high degree of collagen formation, scar shrinkage	[192]
			BC-alginate-gelatin film	Optimal ductility, biocompatibility, increased flexibility, and capacity to absorb water.	[235]
Blood vessels	Connective tissue	Restoration replacement	BC-Fe_3_O_4_NPs magnetic pellicle	Small capillarity blood vessels	[230]
		Biosynthetic blood vessels	BC-polyglycolic acid and expanded polytetrafluorethylene	Biocompatibility (absence of leukocyte activation), apoptotic cell absence, vascularized granulation tissue, and multiple proliferating cells	[208]
		Engineered vessels with anticoagulant property	BC-heparin nanofibrous scaffold	Anticoagulant properties-sulphate groups-enriched BC-heparin hybrid	[236]
		Blood cloth control	BC from nata de coco-kaolin	Topographical properties and malleability of the biomaterial exceed the attraction forces between clotted blood proteins	[237]
		Vascular embolization: interventional therapies	BC-poly-N-isopropyl acrylamide-co-butyl methacrylate nanogel	Thermosensitive injectable biomaterials: expanded to condensed gel state	[238]
Aortic heart valve	Connective tissue	Prospective replacement therapy	BC-PVA hydrogel	Biomimicry: non-linear mechanical properties	[239]
Cartilages	Connective tissue	Replacement, reconstruction	BC-poly(dimethyl acrylamide) double network gel	Meets properties of artificial cartilage; no in vivo tests confirmation	[240]
			BC-PVA composite	Proven elasticity and similar properties to native cartilages	[193]
		Osteochondral defect treatment	Bilayer BC-hydroxyapatite and BC-glycosaminoglycan indice	Accelerated recovery of articular cartilage and subchondral bone in model rats with osteochondral defects	[241]
Bone	Skeletal tissue	Advanced regeneration	BC-bone mesenchymal protein-2 scaffolds	Osteogenesis in rat ectopic models	[242]
		Regeneration, reconstruction	BC-Fisetin scaffold indice	Bone matrix induced biosynthesis	[243]
Gums and Teeth	Connective tissue	Early stages of regeneration	BC-hydroxyapatite-osteogenic growth peptide nanocomposite	Osteoblast differentiation	[244]
		Tooth extraction or transplantation of oral mucosa	Native and oxidized BC-doxycycline	Dental dressings with potential of biodegradability, antimicrobial activity against pathogenic oral bacteria, and suitable drug delivery system	[245]
		Periodontal tissue recovery after dental implants	Inner membrane of BC and external alkali-cellulose (Gengiflex^®^)	Osseo-deficiency treatment: inflammatory response diminished, reduced number of surgical steps, restoration of mouth functions, and aesthetic role	[246]
Eye	Corneal epithelial tissue	Artificial corneal biomaterial	BC/PVA hydrogel	Suitable water content, high visible light transmittance, UV absorbance, proper strength, and thermal properties	[247]
	Retinal pigment epithelium (RPE)	Transplant	Acetylated BC-urinary bladder matrix	Appropriate features as cell carriers in potential RPE transplantation	[248]
Gastro-intestinal level	Connective and epithelial tissues (Simulated gastric and intestinal fluid)	Drug delivery system	BC-polyacrylic acid-bovine albumin (various concentration) hydrogel	Optimization of drug release rate: pH dependent (similar to plain BC membranes)	[249]

Abbreviations: AgNPs—Silver (Ag) nanoparticles, CuO—Copper oxide, Fe_3_O_4_NPs—Iron oxide nanoparticles, PHEMA—Poly(2-hydroxyethyl methacrylate), PHMB—Polyhexamethylene biguanide, PVA—Poly(vinyl alcohol), SOD—Superoxide dismutase, TEMPO-oxidized (2,2,6,6-tetramethyl piperidine oxide), TiO_2_—Titanium Dioxide, ZnO—Zinc oxide.

## Data Availability

No new data were created or analyzed in this study. Data sharing is not applicable to this article.
